# Occurrence and quantification of *Anelloviruses* and *Herpesviruses* in gingival tissue in Chinese Shanghai sub-population

**DOI:** 10.1186/s12903-020-01188-2

**Published:** 2020-07-09

**Authors:** Tian Yu, Shaokun Pan, Yu Zhang, Jun Pei, Jing Liu, Youhua Xie, Xiping Feng

**Affiliations:** 1 Department of Preventive Dentistry, Shanghai Ninth People’s Hospital, College of Stomatology, Shanghai Jiao Tong University School of Medicine, Shanghai, China; 2 National Clinical Research Center for Oral Diseases, Shanghai, China; 3 Shanghai Key Laboratory of Stomatology & Shanghai Research Institute of Stomatology, Shanghai, China; 4 National Clinical Research Center for Aging and Medicine, Huashan Hospital, Fudan University, Shanghai, China; 5 Key Lab of Medical Molecular Virology, School of Basic Medical Sciences, Fudan University, Shanghai, China

**Keywords:** Periodontitis, *Epstein–Barr virus*, *Anelloviruses*, Occurrence, Quantification

## Abstract

**Background:**

*Herpesviruses* and bacteria and their interplay have long been believed to play important roles in the pathogenesis of periodontitis, but other microbial entities in the oral environment might also be involved. *Anelloviruses* are commonly detected in human, including in oral samples. The aim of the present study was to explore the occurrence and co-occurrence of *human cytomegalovirus* (HCMV), *Epstein–Barr virus* (EBV), and *human anelloviruses* (HTTVs) in gingival tissue samples collected from participants recruited in Shanghai, China.

**Methods:**

Gingival tissues were collected from 159 participants (57 with aggressive periodontitis (AP), 59 with chronic periodontitis (CP) and 43 with healthy periodontal status). The presence of HCMV, EBV, *torque teno virus* (TTV), *torque teno mini virus* (TTMV) and *torque teno midi virus* (TTMDV) DNA was detected by nested-PCR. The virus loads were quantified by real-time PCR.

**Results:**

The detection rates of EBV, TTV, TTMV and TTMDV were significantly higher in the AP and CP groups compared to the healthy group (all *P* < 0.01). A statistically significant association was found between EBV, TTV and TTMV virus load and periodontitis (all *P* < 0.05). Participants infected with EBV showed significantly higher infection rates and higher virus loads of TTV and TTMV than the EBV-negative group (all *P* < 0.05). The coexistence rates of EBV and *anelloviruses* and the coexistence of three HTTVs were significantly higher in AP and CP groups (all *P* < 0.01).

**Conclusions:**

Collectively, results obtained in this study suggest that HTTVs and the coexistence of EBV and HTTVs in particular, may be associated with periodontitis. Possible mechanisms of the interaction between *herpesviruses* and *anelloviruses* in the context of periodontitis require further investigation.

## Background

Periodontitis is a chronic, inflammatory oral disease with a high prevalence in Chinese patients [1, 2]. As a multifactorial disease, periodontitis is believed to be caused by interplay among oral microorganisms, the hosts, and environmental factors [3]. Specific bacteria (*Actinobacillus actinomycetemcomitans*, *Porphyromonas gingivalis*, *Tannerella forsythia* and *Treponema denticola)* were considered to be associated with periodontal disease in the traditional theory of periodontal etiology [4]. However, not all clinical symptoms associated with periodontitis can be readily explained by this theory [4]. As a potential risk factor for periodontitis, viruses have been received increasing attention. Viral sequences belonging to including *herpesviruses*, *papilloma viruses*, *human T-lymphotropic virus-111*, and *anelloviruses* have been identified in periodontal lesions [5–7]. Complex interaction between *herpesviruses* and bacteria may underlie the pathogenesis of periodontitis, with *herpesviruses* promoting bacterial adherence, invasion and upgrowth, while bacterial factors facilitating *herpesvirus* colonization and reactivation [8].

Infection by *herpesviruses* has been repeatedly shown to be a potential pathogenic factor for periodontal diseases. Slots [9] summarized the findings of 26 studies on the occurrence of periodontal *herpesviruses* around the world and found that 49% aggressive periodontitis (AP) sites, 40% chronic periodontitis (CP) sites and 3% healthy periodontal sites contained *human cytomegalovirus* (HCMV), while 45% AP sites, 32% CP sites and 7% healthy periodontal sites contained *Epstein–Barr virus* (EBV). A meta-analysis by Botero JE et al. [10] also showed that subgingival HCMV was significantly associated with periodontitis.

*Anelloviruses* are small, non-enveloped viruses with an icosahedral capsid containing the circular, single-strand DNA genome. The first *Anellovirus* was discovered in human samples in 1997, and so far nearly 200 species have been identified in human as well as animals. *Human anelloviruses* (traditionally also called *human torque teno viruses*, HTTVs) currently include members of three genera: *torque teno viruses* (TTV), *torque teno mini virus* (TTMV), and *torque teno midi virus* (TTMDV) [11]. HTTVs have a high prevalence around the world [12–14] and have been identified in diverse biological samples including serum, bone marrow, lung, liver and lymph node [11]. Multiple studies [11–13, 15, 16] have associated HTTVs with hepatitis, liver cancer, infectious gastroenteritis, lymphoma, transfusion borne diseases, colon cancer and severe pneumonia in children, but whether there is a causal link or what role HTTVs might play in the pathogenesis of these diseases remain unclear. In previous studies, members of this group have identified a novel species of TTMV (TTMV-222) in gingival tissue from periodontitis patients [17] and a new TTMV called TTMV-SH in serum from patients with Hodgkin’s lymphoma [18].

Compared to *herpesviruses*, limited information is available regarding the association between HTTVs and periodontitis. Rotundo et al. [7] reported a higher prevalence of TTV in subjects with chronic periodontitis compared to healthy people in Italy. Considering the high prevalence of HTTVs worldwide, it is both interesting and important to assess possible association between HTTVs and periodontitis in other population groups. In addition, the interplay between *herpesviruses* and oral bacteria in the pathogenesis of periodontitis also suggests possible interactions between *herpesviruses* and other oral microorganisms, including HTTVs, which might also be involved in the development of periodontal diseases. For non-oral diseases, Borkosky et al. [19] reported that EBV infections can stimulate TTV replication and the interaction of EBV and TTV might be associated with multiple sclerosis. The aim of the present study is to examine the occurrence and co-occurrence of HTTVs, HCMV and EBV in gingival tissue samples taken from outpatients with aggressive and chronic periodontitis and those with healthy periodontal status in Shanghai, China. The results could not only provide information regarding the gingival occurrence of these viruses in this sampled subpopulation, but might also help reveal possible association of their occurrence or co-occurrence with periodontitis.

## Methods

### Sample collection

People who attended the Ninth People’s Hospital, School of Medicine, Shanghai Jiao Tong University, China from June 2017 to June 2018 were invited to participate in this investigation. All volunteers were healthy without any systemic disease, with at least 20 teeth present (excluding the third molars). The probing depth (PD), clinical attachment loss (CAL), bleeding on probing (BOP), gingival index (GI), and plaque index (PLI) of each volunteer were recorded and X-rays was performed at the initial visit and before surgical treatment. Before the implementation of this epidemiological study, ethical approval (No. 2017195) was obtained from the Institutional Review Board of the Ninth People’s Hospital and this investigation was carried out according to relevant guidelines. Prior to examination and sample collection, written informed consent was obtained from each volunteer.

Fifty-seven participants who were 18–35 years old with aggressive periodontitis, 59 participants who were 36–65 years old with chronic periodontitis and 43 periodontal healthy volunteers who were 18–65 years old were included in this study. In the AP and CP groups, the inclusion criteria were: over 50% teeth with PD ≥ 6 mm, CAL ≥ 3 mm, BOP positive, and alveolar bone resorption detected by pantomography [20]; and exclusion criteria were: systemic disease, HIV infection, heavy cigarette smoking (over 15 cigarettes per day), pregnancy and antibiotic application (within 6 months). For the AP group, at least 6 incisors or first molars involved was required. In the healthy group, participants who had no obvious clinical symptoms of gingivitis or periodontitis and had no teeth with PD > 4 mm, CAL > 1 mm or BOP positive were included. The other inclusion and exclusion criteria were the same as the periodontitis group.

In AP and CP groups, tissue samples obtained during surgical periodontal therapy were the gingival epithelium and connective tissue from internal wall of periodontal pocket facing the sulcus. All participants had been received basic periodontal treatment before surgical periodontal therapy. In the healthy group, biopsy specimens obtained from the sulcular region when healthy teeth were extracted were the gingival epithelium and connective tissue facing the sulcus from periodontal healthy sites. Healthy teeth were extracted for reasons such as orthodontics. To wash away plaque, saliva, and blood, the biopsy specimens were rinsed with sterile phosphate-buffered saline (PBS) at least three times and then immediately put into a clean EP tube (Eppendorf tube) and stored at − 80 °C until further treatment. DNA from weighed samples was extracted by a Takara MiniBEST Viral RNA/DNA Extraction Kit (TaKaRa, Japan) on the basis of the manufacturer’s instructions, and eluted in 100 μL of elution buffer.

### Nested-PCR and real-time PCR assay

Nested-PCR was performed to detect the viruses and the primers of these five viruses were determined according to relevant literature [21, 22]. The primers for EBV were directed to the EBNA2 gene according to Parra B et al. [21]. And the primers for HCMV were directed to the MIE gene according to Parra B et al. [21]. The primers for TTV, TTMV and TTMDV were directed to a highly conserved area located just downstream of the TATA box according to Ninomiya M et al. [22]. The primer sequences and PCR conditions are shown in Table S1 in the supplemental material. The 50-μl PCR reaction mixture contained 2 μl of extracted DNA from the biopsy specimens, 10 pmol of corresponding primers, 25 μl of 2 × PrimerSTAR Max Premix (Takara) and ddH_2_O to reach 50 μl. Positive controls (plasmids containing a targeted fragment of the virus genome), negative controls (DNA extracted from virus negative cells such as Huh7 and 293 T) and blank controls (ddH_2_O) were set in each run. The nested-PCR products were separated by electrophoresis on 3% agarose gels with TAE buffer. The virus-positive subjects were calculated.

Real-time PCR was performed to detect the five virus load and the primers of these five viruses were determined according to relevant literature [19, 22–24]. The primers for EBV were directed to the BALF5 gene according to Borkosky et al. [19]. The primers for HCMV were directed to the US14 gene according to Kubar, A. et al. [24]. The primers for TTV and TTMV were directed to a distinct conserved region that located just upstream of the ORF 2 in TTMV and located within the ORF 2 in TTV according to Moen EM et al. [23]. The primers of Real-time PCR for TTMDV were the same as the inner primers of nested-PCR [22]. Primer sequences and PCR conditions are shown in Table S1 in the supplemental material. Real-time PCR was performed in Roche LightCycler® 480 Instrument II. The 10-μl PCR system consisted of 0.5 μl of extracted DNA from virus-positive samples in nested-PCR, 0.3 μM of each primer, 5 μl of 2 × SuperReal PreMix Plus SYBR Green (TIANGEN) and ddH_2_O to reach 10 μl. Each run contained several negative controls (DNA extracted from virus negative cells such as Huh7 and 293 T), blank controls (ddH_2_O) and positive controls (standard curve). Both controls and samples were assayed in triplicate. Virus DNA could be quantified within a linear range from 10^2^ to 10^9^ copies/μL, as determined by the use of tenfold dilutions of a plasmid standard. The procedures for quantification of copy number and evaluation of intra- and inter- assay precision and reproducibility of the assay have been previously reported [19, 23, 24]. In the authors’ experience, the limit of detection in this study was equivalent to 10^3^ copies per gram of tissue.

### Statistical analyses

In some statistical analyses, the AP and the CP groups were combined into one group called the periodontitis group according to the classification of periodontal diseases in 2018 [25]. All statistical analyses were performed by SPSS software ver. 16.0 (IBM Corporation, Armonk, NY, USA). Descriptive analysis included mean ± standard deviation, median, minimum, maximum and percentages were conducted for all variables. Chi-squared tests and Fisher’s exact tests were applied to analyze differences in sex, the occurrence of the viruses, and the coexistence in three groups. Mann-Whitney U tests and Kruskal-Wallis tests were applied to analyze differences in age, clinical parameters (PD, GI, PLI, and CAL) and the virus loads of the five viruses. The correlation between EBV, TTV and TTMV virus loads was determined by Spearman correlation analysis. The significance level was set at 0.05.

## Results

A total of 159 participants (57 for the aggressive periodontitis group, 59 for the chronic periodontitis group and 43 for the healthy group) were recruited for the study. The age and sex compositions of the three groups are shown in Table 1. There was no significant difference for sex composition, but the age composition was significantly different, with AP and healthy groups consisting of younger subjects compared to CP group. Clinical parameters (PD, GI, PLI, and CAL) were significantly higher in participants with AP or CP than in the healthy group at the initial visit (Table 1).

**Table 1:** Compositions of sex, age and periodontal clinical parameters of three groups

	aggressive periodontitis	chronic periodontitis	healthy	*P*
Sex				0.336^*^
male [n (%)]	31 (54.4)	29 (49.2)	17 (39.5)	
female [n (%)]	26 (45.6)	30 (50.8)	26 (60.5)	
Age (mean ± SD)	29.74 ± 5.357	48.53 ± 8.52	28.81 ± 5.337	< 0.001^**^
PD (mean ± SD)	6.83 ± 1.78	6.17 ± 1.89	1.09 ± 0.78	< 0.001^**^
CAL (mean ± SD)	4.03 ± 0.73	3.98 ± 0.85	0.58 ± 0.51	< 0.001^**^
GI (mean ± SD)	1.92 ± 0.35	2.41 ± 0.28	0.34 ± 0.21	< 0.001^**^
PLI (mean ± SD)	1.58 ± 0.43	2.76 ± 0.31	1.05 ± 0.75	< 0.001^**^

*Chi-squared test

**Kruskal-Wallis test

#Abbreviations**:***n* number of participants, *SD* standard deviation, *PD* the probing depth, *CAL* clinical attachment loss, *GI* gingival index, *PLI* plaque index

Presence of EBV, HCMV and HTTVs sequences in gingival tissues taken from the participants were analyzes using nested-PCR, and positive samples were further analyzed using quantitative real-time PCR (Table 2). Compared to nested-PCR, real-time PCR provided additional quantitative data, but was not as sensitive, possibly due to viral loads below the detection limit of real-time PCR, which is estimated to be equivalent to about 10^3^ copies/g tissue in this work. For occurrence and co-occurrence analyses, nested-PCR results were used. The detection rates of EBV and HCMV in all participants were 36.5 and 6.9%, respectively. The overall occurrences of three HTTVs were higher: 79.2% for TTV, 84.9% for TTMV and 66.7% for TTMDV (Table 2). The detection rates of *herpesviruses* and HTTVs in the three groups are shown in Table 2. The occurrence of EBV was significantly higher (*P* < 0.01) in the AP group (43.9%) and CP group (47.5%) than that in the healthy group (11.6%). Higher positive rates (*P* < 0.01) of the three HTTVs were also detected in the AP group (TTV 93.0%, TTMV 91.2% and TTMDV 75.4%) and the CP group (TTV 84.7%, TTMV 93.2% and TTMDV 78.0%), compared to the healthy group (TTV 53.5%, TTMV 65.1% and TTMDV 39.5%). Between AP and CP groups, however, detection rates of *herpesviruses* and *anelloviruses* were not significantly different (Table S2 in the supplemental material). No statistically significant relationship between detection rate of the studied viruses and sex was identified either (Table S3 in the supplemental material).

**Table 2:** Qualitative and quantitative analysis of *herpesviruses* and *anelloviruses* presence in three groups

	aggressive periodontitis (*n* = 57)	chronic periodontitis (*n* = 59)	healthy (*n* = 43)	*P*	Total (*n* = 159)
**EBV**					
nested-PCR [n + (%)/n-(%)]	25 (43.9) / 32 (56.1)	28 (47.5) / 31 (52.5)	5 (11.6) / 38 (88.4)	< 0.001^*^	58 (36.5) / 101 (63.5)
real-time PCR [n + (%)/n-(%)]	20 (35.1) / 37 (64.9)	26 (44.1) / 33 (55.9)	4 (9.3) / 39 (90.7)	< 0.001^*^	50 (31.4) / 109 (68.6)
virus DNA load Md (Min-Max) (Log10 copies/g)	5.38 (4.41–7.01)	5.89 (5.06–7.31)	5.07 (4.57–5.21)	0.009^**^	5.65 (4.41–7.31)
**HCMV**					
nested-PCR [n + (%)/n-(%)]	3 (5.3) / 54 (94.7)	7 (11.9) / 52 (88.1)	1 (2.3) / 42 (97.7)	0.683^***^	11 (6.9) / 148 (83.1)
real-time PCR [n + (%)/n-(%)]	3 (5.3) / 54 (94.7)	7 (11.9) / 52 (88.1)	1 (2.3) / 42 (97.7)	0.683^***^	11 (6.9) / 148 (83.1)
virus DNA load Md (Min-Max) (Log10 copies/g)	7.34 (7.13–10.14)	9.88 (5.58–10.33)	9.11	0.518^**^	9.33 (5.58–10.33)
**TTV**					
nested-PCR [n + (%)/n-(%)]	53 (93.0) / 4 (7.0)	50 (84.7) / 9 (15.3)	23 (53.5) / 20 (46.5)	< 0.001^*^	126 (79.2) / 33 (20.8)
real-time PCR [n + (%)/n-(%)]	47 (82.5) / 10 (17.5)	48 (81.4) / 11 (18.6)	23 (53.5) / 20 (46.5)	< 0.001^*^	118 (74.2) / 41 (25.8)
virus DNA load Md (Min-Max) (Log10 copies/g)	6.34 (5.00–9.33)	6.76 (5.22–9.39)	6.12 (3.79–8.21)	0.001^**^	6.55 (3.79–9.39)
**TTMV**					
nested-PCR [n + (%)/n-(%)]	52 (91.2) / 5 (8.8)	55 (93.2) / 4 (6.8)	28 (65.1) / 15 (34.9)	< 0.001^*^	135 (84.9) / 24 (15.1)
real-time PCR [n + (%)/n-(%)]	47 (82.5) / 10 (17.5)	51 (86.4) / 8 (13.6)	25 (58.1) / 18 (41.2)	< 0.001^*^	123 (77.4) / 36 (22.6)
virus DNA load Md (Min-Max) (Log10 copies/g)	6.64 (4.90–8.27)	7.05 (4.82–10.20)	6.08 (5.42–8.42)	0.001^**^	6.70 (4.82–10.20)
**TTMDV**					
nested-PCR [n + (%)/n-(%)]	43 (75.4) / 14 (24.6)	46 (78.0) / 13 (22.0)	17 (39.5) / 26 (60.5)	< 0.001^*^	106 (66.7) / 53 (33.3)
real-time PCR [n + (%)/n-(%)]	4 (7.0) / 53 (93.0)	5 (8.5) / 54 (91.5)	3 (7.0) / 40 (93.0)	0.983^***^	12 (7.5) / 147 (92.5)
virus DNA load Md (Min-Max) (Log10 copies/g)	4.79 (4.60–5.41)	4.81 (4.29–5.46)	4.69 (4.62–5.28)	0.851^**^	4.79 (4.29–5.46)

*Chi-squared test

**Kruskal-Wallis test

***Fisher’s exact test

#Abbreviations**:***PCR* polymerase chain reaction, *n* number of participants, *n+* number of positive participants, *n−* number of negative participants, *Md* median, *Min* minimum, *Max* maximum, *HCMV Human cytomegalovirus, EBV Epstein–Barr virus, TTV Torque teno virus, TTMV Torque teno mini virus, TTMDV*: *Torque teno midi virus*

Coexistence of EBV, HCMV and HTTVs in participants was then investigated and the results showed that overall participants infected with EBV had significantly higher infection rates (*P* < 0.05) of HTTVs (TTV 89.1%, TTMV 93.1% and TTMDV 79.3%) compared to EBV-negative participants (TTV 73.3%, TTMV 80.2% and TTMDV 59.4%) (Table 3). The detection rates of *herpesvirus* and HTTVs coexistence in the three groups are shown in Table 4 and Table S4 in the supplemental material. When the three groups were compared, coexistence of EBV and any of the three HTTVs were significantly higher (*P* < 0.01) in AP and CP groups than in the healthy group (Table 4). The coexistence among HTTVs were also significantly higher in the AP and CP groups (*P* < 0.01) (Table 4). Due to its low occurrence, especially in healthy group, coexistence of HCMV with other viruses showed no significant differences among the three groups (Table 4).

**Table 3:** Qualitative and quantitative analysis of *anelloviruses* presence in EBV positive and negative groups

	EBV positive (*n* = 58)	EBV negative (*n* = 101)	*P*
**TTV**			
nested-PCR [n + (%)/n-(%)]	52 (89.7) / 6 (10.3)	74 (73.3) / 27 (26.7)	0.014^*^
virus DNA load Md (Min-Max) (Log_10_ copies/g)	6.68 (4.95–9.39)	6.40 (3.79–8.26)	0.023^**^
**TTMV**			
nested-PCR [n + (%)/n-(%)]	54 (93.1) / 4 (6.9)	81 (80.2) / 20 (19.8)	0.029^*^
virus DNA load Md (Min-Max) (Log_10_ copies/g)	6.95 (5.05–10.20)	6.40 (4.82–9.84)	0.004^**^
**TTMDV**			
nested-PCR [n + (%)/n-(%)]	46 (79.3) / 12 (20.7)	60 (59.4) / 41 (40.6)	0.01^*^
virus DNA load Md (Min-Max) (Log_10_ copies/g)	4.79 (4.29–5.46)	4.69 (4.62–5.28)	0.782^**^

*Chi-squared test

**Mann-Whitney U test

#Abbreviations**:***PCR* polymerase chain reaction, *Md* median, *Min* minimum, *Max* maximum, *n* number of participants, *n+* number of positive participants, *n−* number of negative participants, *EBV Epstein–Barr virus, TTV Torque teno virus, TTMV Torque teno mini virus, TTMDV Torque teno midi virus*

**Table 4:** Coexistence of *herpesviruses* and *anelloviruses* in three groups

Coexistence nested-PCR	aggressive periodontitis positive n (%)	chronic periodontitis positive n (%)	healthy positive n (%)	*P*
EBV + HCMV	1 (1.8)	5 (8.5)	0	0.804^**^
EBV + TTV	24 (42.1)	25 (42.4)	3 (7)	<0.001^*^
EBV + TTMV	22 (38.6)	27 (45.8)	5 (11.6)	0.001^*^
EBV + TTMDV	19 (33.3)	25 (42.4)	2 (4.7)	<0.001^*^
HCMV+TTV	3 (5.3)	6 (10.2)	1 (2.3)	0.644^**^
HCMV+TTMV	3 (5.3)	7 (11.9)	1 (2.3)	0.683^**^
HCMV+TTMDV	3 (5.3)	7 (11.9)	1 (2.3)	0.683^**^
TTV + TTMV	50 (87.7)	48 (81.4)	17 (39.5)	<0.001^*^
TTV + TTMDV	42 (73.7)	41 (69.5)	14 (32.6)	<0.001^*^
TTMV+TTMDV	42 (73.7)	45 (76.3)	15 (34.9)	<0.001^*^
TTV + TTMV+TTMDV	41 (71.9)	41 (69.5)	13 (30.2)	<0.001^*^

*Chi-squared test

**Fisher’s exact test

#Abbreviations**:***PCR* polymerase chain reaction, *n* number of participants, *HCMV Human cytomegalovirus, EBV Epstein–Barr virus, TTV Torque teno virus, TTMV Torque teno mini virus, TTMDV Torque teno midi virus*

Quantification results obtained using real-time PCR showed that EBV virus loads ranged from 4.41 to 7.32 log_10_ copies/g, HCMV loads ranged from 5.58 to 10.33 log_10_ copies/g, TTV loads ranged from 3.79 to 9.39 log_10_ copies/g, TTMV loads ranged from 4.81 to 10.20 log_10_ copies/g and TTMDV loads ranged from 4.29 to 5.46 log_10_ copies/g (Table 2). Notably, a predominant majority of TTMDV-positive samples had viral loads below the detection limit. The virus loads of EBV, TTV and TTMV showed significant differences among the AP, CP and healthy groups (*P* < 0.05) (Table 2), while TTV and TTMV virus loads in the CP group were significantly higher than those in the AP group (Table S2 in the supplemental material). In addition, among all participants, EBV-positive participants had higher TTV and TTMV virus loads compared to EBV-negative participants (*P* < 0.05) (Table 3). A positive correlation between the virus loads of EBV and TTMV and the virus loads of TTV and TTMV was also identified by Spearman correlation analysis (*P* < 0.05) (Table 5).

**Table 5:** Correlation between virus loads of EBV, TTV and TTMV (log_10_ copies/g)

	spearman correlation coefficient	***P***
EBV-TTV	0.139	0.325^*^
EBV-TTMV	0.372	0.006^*^
TTV-TTMV	0.484	< 0.001^*^

*Spearman correlation analysis

#Abbreviations**:***EBV Epstein–Barr virus*, *TTV Torque teno virus*, *TTMV Torque teno mini virus*

The differences between the periodontitis group and healthy group in the detection rates and the virus loads of *herpesvirus* and *anelloviruses* are shown in Table S5 and S6 in the supplemental material. The detection rates and the virus loads of EBV, TTV and TTMV were significantly higher in the periodontitis group. And the periodontitis group showed higher coexistence rates of EBV and HTTVs.

## Discussion

Periodontitis was found to have a higher prevalence in Chinese than before and became one of the major reasons for tooth loss [1, 2]. According to the classification of periodontal diseases in 2018 [25], chronic and aggressive periodontitis are now grouped under a single category (“Periodontitis”). But this study was designed and initiated before the publication of the new scheme. Stringent inclusion/exclusion criteria based on the traditional AP/CP classification were implemented in the hope of identifying possible AP- and CP-associated differences. So the classification of periodontal diseases in 1999 [20] was used in this study. According to the new classification in 2018, participants in AP and CP groups could be diagnosed as generalized periodontitis with stage III or stage IV. The results (Table S5 and S6 in the supplemental material) were similar to the outcomes of AP, CP and healthy groups.

Previous reports [6, 9, 26–28] have repeatedly suggested that EBV and HCMV are involved in the pathogenesis of periodontal disease in various population groups. In the current study, it was found that the occurrence of EBV was 43.9% in the AP group, 47.5% in the CP group and significantly higher than the occurrence of 11.6% in the healthy group (*P* < 0.05). Moreover, the EBV virus loads were significantly higher in the AP and CP groups (*P* < 0.05). These results are consistent with earlier reports about EBV in periodontitis [6, 9, 26, 29, 30]. However, the occurrence of HCMV was 5.3 and 11.9% in the AP and CP groups in this study, lower than what many previous studies have reported [31–33]. For instance, using the same nested-PCR primers, Parra B et al. reported occurrences of EBV and HCMV in periodontitis patients to be 30 and 60% respectively [21]. In addition, no significant differences were observed between AP and CP groups with regard to detection rates of either EBV or HCMV, whereas previously, Slots [9] reported higher occurrence of *herpesviruses* in patients with AP compared to patients with CP. These apparent discrepancies could be the result of differences in population studied and/or sampling technique used. Dawson et al. [34] summarized HCMV detection rates in studies that used samples from single site of periodontitis and found that detection rates varied from 0.5 to 59%. These authors suggested that [34] lower detection might be due to the fact that HCMV were in a latent state and would contribute to the progression of periodontitis by reactivation, which might also apply to some AP/CP subjects in this study. Furthermore, HCMV mainly infects periodontal monocytes and macrophages [35] that might be distributed unevenly in gingival tissues [36]. In this study, gingival tissue sample was taken from a single site on each participant, and consequently may not truly represent the HCMV infection status of gingival. Many previous studies [6, 9, 26–28, 34, 37] detected viruses in samples of saliva, subgingival plaque, serum or gingival tissues. Due to the difficulty of sampling, the authors didn’t obtain other samples from the same participants in this study, which was the limitation of this investigation. Clearly, for detection of *herpesviruses* in the context of periodontitis, low detection rates such as observed for HCMV in this work would necessarily require further testing on additional samples for confirmation.

Many studies have reported that *anelloviruses* frequently and ubiquitously infect humans [12–14]. Although the detection rates of different types of HTTVs in various areas were quite different, it is generally believed that HTTVs can be detected in more than 50% of the population [11]. The findings of the present study showed overall high detection rates of TTV, TTMV and TTMDV, and the detection rates in the AP and CP groups were significantly higher than the healthy group. Moreover, the CP group had significantly higher TTV and TTMV virus loads than the healthy group (*P* < 0.05). The high detection rates of all three HTTVs are consistent with prevalence data in the Middle East obtained using the same nested PCR primers as used in this study [12]. Also using the same real-time PCR primers, Garcia-Alvarez M et al. reported that the prevalence and the virus loads of TTV and TTMV were markedly elevated in HIV/HCV-coinfected patients and HIV-monoinfected patients than the control group [16]. The finding of high TTV occurrence and virus loads in gingival tissues agrees with results from the study by Rotundo et al. in periodontitis patients [7]. In addition, this study identified higher TTV and TTMV virus loads in CP group compared to AP group. TTMDV virus loads in most samples that were positive by nested-PCR were apparently too low to be quantified using real-time PCR in this work. Due to the rare reporting on real-time PCR quantification of TTMDV in the literature, it is as yet unclear whether this phenomenon is specific to the studied cohort. Nevertheless, higher detection rates of HTTVs and higher virus loads of TTV and TTMV observed in AP and CP patients, as well as differences in TTV and TTMV virus loads between AP and CP groups, suggest a possible association of HTTVs with periodontitis that warrants further studies.

Coexistence of multiple *anelloviruses* seems to be a common event [16, 38]. However, most of the reports investigated only one or two of the three HTTVs. Studies that explored all of three viruses are rare. Al-Qahtani et al. [12] found that the detection rates of TTV, TTMV, TTMDV and their coexistence were high in blood samples. In this study, the positive rates of the coexistence of the three HTTVs in the AP and CP groups (71.9 and 69.5%) were significantly higher than in healthy group (30.2%) (Table 4). The TTV and TTMV virus loads also displayed positive correlation (*P* < 0.05). This suggests potential association between different HTTVs in the context of periodontitis, which also warrants further studies.

Because the oral cavity is an open environment and colonized by a large number of microorganisms, periodontitis is believed to be multifactorial. The relationship between EBV and other microbial entities in the context of periodontitis has been explored by many researchers. Slots et al. [39] summarized the recent studies on the periodontal treatment and found that the use of systemic chemotherapy and inexpensive antiseptics against bacterial pathogens and periodontal *herpesviruses* may help to arrest active periodontitis. Jakovljevic A et al. [40] hypothesize that EBV infection could stimulate the overproduction of reactive oxygen species (ROS) to induce periapical bone resorption in apical periodontitis. Makino K et al. [41] found that n-butyric acid produced by *P. endodontalis* could reactivate latent EBV by inducing the expression of BZLF-1 mRNA and ZEBRA protein. However, very few studies have reported about the association between EBV and *anelloviruses*. Sriraman et al. [42] found that TTV, EBV and HCMV could be detected in cardiac patients with atherosclerosis and coexisting chronic periodontitis but there was no significant association of the three viruses. Borkosky et al. [19] found that EBV infections can stimulate TTV replication and the interaction of EBV and TTV may be associated with multiple sclerosis. In the present study, EBV detection rates and virus loads in AP and CP patients were significantly higher compared to the healthy group (Table 2), echoing previous studies linking EBV to periodontitis. Against this background, this study further identified higher detection rates and virus loads of TTV and TTMV in the EBV positive subjects compared to EBV negative subjects (*P* < 0.05) (Tables 3), and in addition, higher co-occurrence of EBV with TTV, TTMV and TTMDV in both AP and CP patients compared to healthy participants (Table 4). These results suggest a possible association of EBV with HTTVs, especially in the context of periodontitis. Whether there is an underlying interplay between EBV and HTTVs in such cases, as shown by the findings of Borkosky et al. [22], would be an interesting direction to pursue in future research.

The possible pathogenic mechanism of HTTVs has been a highly controversial question ever since their discovery, due in no small part to the lack of suitable cell lines to culture viruses. Yokoyama et al. [43] used transgenic mouse model to show that TTV ORF1 protein appeared to affect the differentiation of kidney epithelial cells. Galmès et al. [15] found that TTMV-LY could infect lung alveolar epithelial cells and induce the expression of pro-inflammatory cytokines to regulate the innate immune response of lung tissue. Results obtained in this study indicate that potential pathogenesis by HTTVs might need to be studied in the presence of commonly found co-infecting viruses such as EBV.

## Conclusions

In summary, this study showed the high occurrence of EBV, TTV, TTMV and TTMDV in aggressive and chronic periodontitis patients compared to periodontal healthy controls recruited in Shanghai, China. Quantification by real-time PCR also identified higher EBV, TTV and TTMV virus loads in periodontitis gingival samples. Furthermore, higher co-occurrence of EBV and HTTVs was observed in periodontitis patients. Collectively, these results suggest that HTTVs and coexistence of EBV and HTTVs in particular, may be associated with periodontitis. Possible mechanisms of the interaction between *herpesviruses* and *anelloviruses* in the context of periodontitis require further investigation.

## Supplementary information

**Additional file 1: Table S1.** Primer sequences and PCR conditions of *herpesviruses* and *anelloviruses.***Table S2.** Qualitative and quantitative analysis of *herpesviruses* and *anelloviruses* presence in aggressive periodontitis and chronic periodontitis groups. **Table S3.** Detection rates of *herpesviruses* and *anelloviruses* by sex in periodontitis and healthy groups. **Table S4.** Association between TTV, TTMV, and TTMDV in periodontitis and healthy groups. **Table S5.** Qualitative and quantitative analysis of *herpesviruses* and *anelloviruses* presence in periodontitis and healthy groups. **Table S6.** Coexistence of *herpesviruses* and *anelloviruses* in periodontitis and healthy groups.

## Data Availability

The datasets during and/or analysed during the current study available from the corresponding author on reasonable request.

## Abbreviations

PCR: Polymerase chain reaction
n: number of participants
n+: number of positive participants
n−: number of negative participants.
Md: Median
Min: Minimum
Max: Maximum
HCMV: Human cytomegalovirus
EBV: Epstein–Barr virus
TTV: Torque teno virus
TTMV: Torque teno mini virus
TTMDV: Torque teno midi virus
HTTVs: *Human torque teno viruses*
SD: Standard deviation
PD: The probing depth
CAL: Clinical attachment loss
GI: Gingival index
PLI: Plaque index
