# Supplementary material for: Occurrence and quantification of Anelloviruses and Herpesviruses in gingival tissue in Chinese Shanghai sub-population
Source: BMC Oral Health. 2020 Jul 9;20:196. doi: 10.1186/s12903-020-01188-2 (PMC7346523; doi:10.1186/s12903-020-01188-2)
Supplement: Supplementary file 1 — Additional file 1: Table S1. Primer sequences and PCR conditions of herpesviruses and anelloviruses.Table S2. Qualitative and quantitative analysis of herpesviruses and anelloviruses presence in aggressive periodontitis and chronic periodontitis groups. Table S3. Detection rates of herpesviruses and anelloviruses by sex in periodontitis and healthy groups. Table S4. Association between TTV, TTMV, and TTMDV in periodontitis and healthy groups. Table S5. Qualitative and quantitative analysis of herpesviruses and anelloviruses presence in periodontitis and healthy groups. Table S6. Coexistence of herpesviruses and anelloviruses in periodontitis and healthy groups. [file 12903_2020_1188_MOESM1_ESM.docx]

**Table S1. Primer sequences and PCR conditions of *herpesviruses* and *anelloviruses***

| Viruses | Polarity | Primer sequence (5'-3') | Amplicon length (bp) | Reference | PCR conditions | | | | |
| --- | --- | --- | --- | --- | --- | --- | --- | --- | --- |
| **nested-PCR primers** |  |  |  |  | pre-  denaturation | 30 cycles | | | final extension |
| EBV outer primer | Sense | AGGGATGCCTGGACACAAGA | NA | [21] | 94℃ | 94℃ | 55℃ | 72℃ | 72℃ |
|  | Antisense | TGGTGCTGCTGGTGGCAA |  |  | 5min | 30s | 30s | 40s | 5min |
| EBV inner primer | Sense | TCTTGATAGGGATCCGCTAGGATA | 497 | [21] | 94℃ | 94℃ | 55℃ | 72℃ | 72℃ |
|  | Antisense | ACCGTGGTTCTGGACTATTCGGATC |  |  | 5min | 30s | 30s | 40s | 5min |
| HCMV outer primer | Sense | CAGACACAGTGTCCTCCCGCTCCTC | NA | [21] | 94℃ | 94℃ | 55℃ | 72℃ | 72℃ |
|  | Antisense | CCTAGTGTGGATGACCTACGGGCCA |  |  | 5min | 30s | 30s | 20s | 5min |
| HCMV inner primer | Sense | CAGACACAGTGTCCTCCCGCTCCTC | 136 | [21] | 94℃ | 94℃ | 55℃ | 72℃ | 72℃ |
|  | Antisense | CCAGAGTCCCCTGTACCCGC |  |  | 5min | 30s | 30s | 20s | 5min |
| TTV,TTMV and TTMDV outer primer | Sense | ACWKMCGAATGGCTGAGTTT | NA | [22] |  |  |  |  |  |
|  | Sense | RGTGRCGAATGGYWGAGTTT |  |  | 94℃ | 94℃ | 55℃ | 72℃ | 72℃ |
|  | Antisense | CCCKWGCCCGARTTGCCCCT |  |  | 5min | 30s | 30s | 20s | 5min |
|  | Antisense | AYCTWGCCCGAATTGCCCCT |  |  |  |  |  |  |  |
| TTV inner primer | Sense | ACWKMCGAATGGCTGAGTTT | 112-117 | [22] | 94℃ | 94℃ | 55℃ | 72℃ | 72℃ |
|  | Sense | RGTGRCGAATGGYWGAGTTT |  |  | 5min | 30s | 30s | 40s | 5min |
|  | Antisense | CCCCTTGACTBCGGTGTGTAA |  |  |  |  |  |  |  |
| TTMV inner primer | Sense | TTTATGCYGCYAGACGRAGA | 70-72 | [22] |  |  |  |  |  |
|  | Sense | TTTAYCMYGCCAGACGGAGA |  |  | 94℃ | 94℃ | 55℃ | 72℃ | 72℃ |
|  | Sense | TTTATGCCGCCAGACGRAGG |  |  | 5min | 30s | 30s | 20s | 5min |
|  | Antisense | CTCACCTYSGGCWCCCGCCC |  |  |  |  |  |  |  |
| TTMDV inner primer | Sense | SGABCGAGCGCAGCGAGGAG | 88 | [22] | 94℃ | 94℃ | 55℃ | 72℃ | 72℃ |
|  | Antisense | GCCCGARTTGCCCCTAGACC |  |  | 5min | 30s | 30s | 40s | 5min |
| **Realtime PCR primers** |  |  |  |  | pre-  denaturation | 45 cycles | | | melting curve |
| EBV-Realtime PCR | Sense | CTTTGGCGCGGATCCTC | 90 | [19] | 95℃ | 95℃ | 60℃ | 72℃ |  |
|  | Antisense | AGTCCTTCTTGGCTAGTCTGTTGAC |  |  | 15min | 10s | 20s | 20s |  |
| HCMV-Real-time PCR | Sense | TGAGCCCGGCGGTGGT | 84 | [24] | 95℃ | 95℃ | 60℃ | 72℃ |  |
|  | Antisense | AGCTCACCGATCACAGACAC |  |  | 15min | 10s | 20s | 20s |  |
| TTV-Realtime PCR | Sense | GTTTTCTACGCCCGTCC | 105 | [23] |  |  |  |  |  |
|  | Sense | GTTTTCCACGCCCGTCC |  |  | 95℃ | 95℃ | 60℃ | 72℃ |  |
|  | Antisense | CCTTGACTCCGGTGTGTAA |  |  | 15min | 10s | 20s | 20s |  |
|  | Antisense | CCTTGACTKCGGTGTGTAA |  |  |  |  |  |  |  |
| TTMV-Realtime PCR | Sense | AGTTTATGCCGCCAGACG | 94 | [23] | 95℃ | 95℃ | 60℃ | 72℃ |  |
|  | Antisense | CCCTAGACTTCGGTGGTTTC |  |  | 15min | 10s | 20s | 20s |  |
| TTMDV-Realtime PCR | Sense | SGABCGAGCGCAGCGAGGAG | 88 | [22] | 95℃ | 95℃ | 60℃ | 72℃ |  |
|  | Antisense | GCCCGARTTGCCCCTAGACC |  |  | 15min | 10s | 20s | 20s |  |

*W denotes A or T, K denotes G or T, M denotes A or C, R denotes A or G, Y denotes C or T, S denotes C or G, and B denotes C, G, or T.

#Abbreviations**:** PCR: polymerase chain reaction; HCMV: *Human cytomegalovirus*; EBV: *Epstein–Barr virus*; TTV: *Torque teno virus*; TTMV: *Torque teno mini virus*; TTMDV: *Torque teno midi virus*.

**Table S2. Qualitative and quantitative analysis of *herpesviruses* and *anelloviruses* presence in aggressive periodontitis and chronic periodontitis groups**

|  | aggressive periodontitis (n=57) | chronic periodontitis (n=59) | *P* |
| --- | --- | --- | --- |
| **EBV** |  |  |  |
| nested-PCR [n+(%)/n-(%)] | 25(43.9) / 32(56.1) | 28(47.5) / 31(52.5) | 0.697^*^ |
| real-time PCR [n+(%)/n-(%)] | 20(35.1) / 37(64.9) | 26(44.1) / 33(55.9) | 0.323^*^ |
| virus DNA load Md(Min-Max) (Log_10_ copies/g) | 5.38(4.41-7.01) | 5.89(5.06-7.31) | 0.101^**^ |
| **HCMV** |  |  |  |
| nested-PCR [n+(%)/n-(%)] | 3(5.3) / 54(94.7) | 7(11.9) / 52(88.1) | 0.350^*^ |
| real-time PCR [n+(%)/n-(%)] | 3(5.3) / 54(94.7) | 7(11.9) / 52(88.1) | 0.350^*^ |
| virus DNA load Md(Min-Max) (Log_10_ copies/g) | 7.34(7.13-10.14) | 9.88(5.58-10.33) | 0.305^**^ |
| **TTV** |  |  |  |
| nested-PCR [n+(%)/n-(%)] | 53(93.0) / 4(7.0) | 50(84.7) / 9(15.3) | 0.16^*^ |
| real-time PCR [n+(%)/n-(%)] | 47(82.5) / 10(17.5) | 48(81.4) / 11(18.6) | 0.878^*^ |
| virus DNA load Md(Min-Max) (Log_10_ copies/g) | 6.34(5.00-9.33) | 6.76(5.22-9.39) | 0.002^**^ |
| **TTMV** |  |  |  |
| nested-PCR [n+(%)/n-(%)] | 52(91.2) / 5(8.8) | 55(93.2) / 4(6.8) | 0.688^*^ |
| real-time PCR [n+(%)/n-(%)] | 47(82.5) / 10(17.5) | 51(86.4) / 8(13.6) | 0.553^*^ |
| virus DNA load Md(Min-Max) (Log_10_ copies/g) | 6.64(4.90-8.27) | 7.05(4.82-10.20) | 0.009^**^ |
| **TTMDV** |  |  |  |
| nested-PCR [n+(%)/n-(%)] | 43(75.4) / 14(24.6) | 46(78.0) / 13(22.0) | 0.747^*^ |
| real-time PCR [n+(%)/n-(%)] | 4(7.0) / 53(93.0) | 5(8.5) / 54(91.5) | 0.999^***^ |
| virus DNA load Md(Min-Max) (Log_10_ copies/g) | 4.79(4.60-5.41) | 4.81(4.29-5.46) | 0.624^**^ |

*Chi-squared test

**Mann-Whitney U test

***Fisher’s exact test

#Abbreviations**:** PCR: polymerase chain reaction; Md: median; Min: minimum; Max: maximum; n: number of participants; n+: number of positive participants; n−: number of negative participants; HCMV: *Human cytomegalovirus*; EBV: *Epstein–Barr virus*; TTV: *Torque teno virus*; TTMV: *Torque teno mini virus*; TTMDV: *Torque teno midi virus*.

**Table S3. Detection rates of *herpesviruses* and *anelloviruses* by sex in periodontitis and healthy groups**

|  | male | female | *P* |
| --- | --- | --- | --- |
| nested-PCR | positive n(%) | positive n(%) |  |
| EBV |  |  |  |
| periodontitis | 32(53.3) | 21(37.5) | 0.087^*^ |
| healthy | 3(17.6) | 2(7.7) | 0.319^**^ |
| HCMV |  |  |  |
| periodontitis | 7(11.7) | 3(5.4) | 0.226^**^ |
| healthy | 0 | 1(2.3) | N/A |
| TTV |  |  |  |
| periodontitis | 56(93.3) | 47(83.9) | 0.109^*^ |
| healthy | 10(58.8) | 13(50) | 0.571^*^ |
| TTMV |  |  |  |
| periodontitis | 55(91.6) | 52(92.9) | 0.811^*^ |
| healthy | 12(70.6) | 16(61.5) | 0.543^*^ |
| TTMDV |  |  |  |
| periodontitis | 50(83.3) | 46(82.1) | 0.856^*^ |
| healthy | 8(47.1) | 10(38.5) | 0.576^*^ |

*Chi-squared test

**Fisher’s exact test

#Abbreviations**:** PCR: polymerase chain reaction; n: number of participants; HCMV: *Human cytomegalovirus*; EBV: *Epstein–Barr virus*; TTV: *Torque teno virus*; TTMV: *Torque teno mini virus*; TTMDV: *Torque teno midi virus*.

**Table S4. Association between TTV, TTMV, and TTMDV in periodontitis and healthy groups**

| nested-PCR |  | TTMV n (%) | *P* | TTMDV n (%) | *P* |
| --- | --- | --- | --- | --- | --- |
| periodontitis(n=116) | TTV | 98(84.5) | ＜0.01^*^ | 83(71.6) | 0.016^*^ |
|  | TTMDV | 87(75) | ＜0.01^*^ | - | - |
| healthy(n=43) | TTV | 17(39.5) | 0.194^*^ | 14(32.6) | 0.01^*^ |
|  | TTMDV | 15(34.9) | ＜0.01^*^ | - | - |

*Chi-squared test

#Abbreviations**:** PCR: polymerase chain reaction; n: number of participants; TTV: *Torque teno virus*; TTMV: *Torque teno mini virus*; TTMDV: *Torque teno midi virus*.

**Table S5. Qualitative and quantitative analysis of *herpesviruses* and *anelloviruses* presence in periodontitis and healthy groups**

|  | periodontitis (n=116) | healthy (n=43) | *P* |
| --- | --- | --- | --- |
| **EBV** |  |  |  |
| nested-PCR [n+(%)/n-(%)] | 53(45.7)/ 63(54.3) | 5(11.6) / 38(88.4) | ＜0.001^*^ |
| real-time PCR [n+(%)/n-(%)] | 46(39.7) / 70(60.3) | 4(9.3) / 39(90.7) | ＜0.001^*^ |
| virus DNA load Md(Min-Max) (Log_10_ copies/g) | 5.732(4.41-7.32) | 5.07(4.57-5.21) | 0.009^**^ |
| **HCMV** |  |  |  |
| nested-PCR [n+(%)/n-(%)] | 10(8.6) / 106(91.4) | 1(2.3) / 42(97.7) | 0.299^***^ |
| real-time PCR [n+(%)/n-(%)] | 10(8.6) / 106(91.4) | 1(2.3) / 42(97.7) | 0.299^***^ |
| virus DNA load Md(Min-Max) (Log_10_ copies/g) | 9.60(5.58-10.33) | 9.11 | 0.752 |
| **TTV** |  |  |  |
| nested-PCR [n+(%)/n-(%)] | 103(88.8) / 13(11.2) | 23(53.5) / 20(46.5) | ＜0.001^*^ |
| real-time PCR [n+(%)/n-(%)] | 95(81.9) / 21(18.1) | 23(53.5) / 20(46.5) | ＜0.001^*^ |
| virus DNA load Md(Min-Max) (Log_10_ copies/g) | 6.54(5.00-9.39) | 6.12(3.79-8.21) | 0.023^**^ |
| **TTMV** |  |  |  |
| nested-PCR [n+(%)/n-(%)] | 107(92.2) / 9(7.8) | 28(65.1) / 15(34.9) | ＜0.001^*^ |
| real-time PCR [n+(%)/n-(%)] | 98(84.5) / 18(15.5) | 25(58.1) / 18(41.2) | ＜0.001^*^ |
| virus DNA load Md(Min-Max) (Log_10_ copies/g) | 6.80(4.82-10.20) | 6.08(5.42-8.42) | 0.009^**^ |
| **TTMDV** |  |  |  |
| nested-PCR [n+(%)/n-(%)] | 89(76.7) / 27(23.3) | 17(39.5) / 26(60.5) | ＜0.001^*^ |
| real-time PCR [n+(%)/n-(%)] | 9(7.7) / 107(92.2) | 3(7.0) / 40(93.0) | 0.754^***^ |
| virus DNA load Md(Min-Max) (Log_10_ copies/g) | 4.79(4.29-5.45) | 4.81(4.29-5.46) | 0.782^**^ |

*Chi-squared test

**Mann-Whitney U test

***Fisher’s exact test

#Abbreviations**:** PCR: polymerase chain reaction; Md: median; Min: minimum; Max: maximum; n: number of participants; n+: number of positive participants; n−: number of negative participants; HCMV: *Human cytomegalovirus*; EBV: *Epstein–Barr virus*; TTV: *Torque teno virus*; TTMV: *Torque teno mini virus*; TTMDV: *Torque teno midi virus*.

**Table S6. Coexistence of *herpesviruses* and *anelloviruses* in periodontitis and healthy groups**

| Coexistence  nested-PCR | Periodontitis  positive n(%) | Healthy  positive n(%) | *P* |
| --- | --- | --- | --- |
| EBV+HCMV | 6(5.2%) | 0 | 0.192^**^ |
| EBV+TTV | 49(42.2%) | 3(7%) | ＜0.001^*^ |
| EBV+TTMV | 49(42.2%) | 5(11.6%) | ＜0.001^*^ |
| EBV+TTMDV | 44(37.9%) | 2(4.7%) | ＜0.001^**^ |
| HCMV+TTV | 9(7.8%) | 1(2.3%) | 0.289^**^ |
| HCMV+TTMV | 10(8.6%) | 1(2.3%) | 0.291^**^ |
| HCMV+TTMDV | 10(8.6%) | 1(2.3%) | 0.291^**^ |
| TTV+TTMV | 98(84.5%) | 17(39.5%) | ＜0.001* |
| TTV+TTMDV | 83(71.6%) | 14(32.6%) | ＜0.001^*^ |
| TTMV+TTMDV | 87(75%) | 15(34.9%) | ＜0.001^*^ |
| TTV+TTMV+TTMDV | 82(70.7%) | 13(30.2%) | ＜0.001^*^ |

*Chi-squared test

**Fisher’s exact test

#Abbreviations**:** PCR: polymerase chain reaction; n: number of participants; HCMV: *Human cytomegalovirus*; EBV: *Epstein–Barr virus*; TTV: *Torque teno virus*; TTMV: *Torque teno mini virus*; TTMDV: *Torque teno midi virus*.
